# Interleukin-2 alters distribution of CD144 (VE-cadherin) in endothelial cells

**DOI:** 10.1186/1479-5876-12-113

**Published:** 2014-05-06

**Authors:** Dae Won Kim, Andrew Zloza, Joseph Broucek, Jason M Schenkel, Carl Ruby, Georges Samaha, Howard L Kaufman

**Affiliations:** 1 Department of General Surgery, Rush University Medical Center, Chicago, IL, USA; 2 Departments of Immunology/Microbiology and Internal Medicine, Rush University Medical Center, Chicago, IL, USA; 3 Center for Immunology, Department of Microbiology, University of Minnesota, Minneapolis, MN, USA; 4 Rutgers Cancer Institute of New Jersey, Rutgers University, 195 Little Albany Street, Room 2007, New Brunswick, NJ 08901, USA

**Keywords:** CD144, Interleukin-2, VE-cadherin, Vascular leak syndrome

## Abstract

**Background:**

High-dose IL-2 (HDIL2) is approved for the treatment of metastatic melanoma and renal cell carcinoma, but its use is limited in part by toxicity related to the development of vascular leak syndrome (VLS). Therefore, an understanding of the mechanisms that underlie the initiation and progression of HDIL2-induced increases in endothelial cell (EC) permeability leading to VLS are of clinical importance.

**Methods:**

We established a novel ex vivo approach utilizing primary human pulmonary microvascular ECs to evaluate EC barrier dysfunction in response to IL-2.

**Results:**

Complementary in vitro studies using exogenous IL-2 and ex vivo studies using serum from patients treated with IL-2 demonstrate that HDIL2 induces VLS through CD144 (vascular endothelial (VE)-cadherin) redistribution.

**Conclusions:**

These findings provide new insight into how IL-2 induces VLS and identifies VE-cadherin as a potential target for preventing IL-2-related VLS.

## Background

High-dose IL-2 (HDIL2) is an approved immunotherapy for patients with metastatic melanoma and renal cell carcinoma with durable objective responses observed in 17-20% [1,2]. The major toxicity related to HDIL2 treatment is the development of vascular leak syndrome (VLS) characterized by increased vascular permeability leading to decreased end-organ perfusion and, in severe cases, pulmonary and cardiovascular failure [2-5]. The mechanisms that underlie the development of vascular leak during HDIL2 therapy are not well understood, but it has been hypothesized that such mechanisms involve the direct effects of IL-2 on endothelial cells (ECs) [6-8]. Currently, no clinical strategies are available for the prevention of VLS in HDIL2-treated patients.

Investigation into the pathogenesis of vascular leak syndrome is complicated by a lack of appropriate animal or ex vivo models that accurately replicate human endothelial tissue. The goal of this study was to establish an ex vivo endothelial cell model for examining the mechanism of endothelial cell dysfunction during HDIL2 immunotherapy using primary human pulmonary microvascular ECs. Understanding the causes of vascular leak syndrome and EC-mediated injury during HDIL2 therapy may help identify novel therapeutic targets to limit the adverse effects in these patients while maintaining the direct effects on immune cells and, thus, preserve the therapeutic benefit of HDIL2 treatment in patients with cancer.

## Methods

### Patient sample acquisition

Eight patients with metastatic melanoma or renal cell carcinoma treated with high-dose bolus IL-2 between September 2003 and July 2005 were eligible for participation (Table 1). The clinical protocol was approved by the Institutional Review Board, and written informed consent was obtained from all patients prior to the initiation of the study. Patients also met the institutional standards for high-dose IL-2 administration. High-dose, bolus IL-2 was administered at 600,000 IU/kg every 8 hrs up to 15 maximum doses or until irreversible grade 3 adverse events occurred. Whole blood was collected prior to the initiation of IL-2 treatment and within eight hours after the fourth dose of each cycle of IL-2 therapy. Clinical data were collected on each patient by chart review and blood pressure less than 90 mmHg systolic was recorded as hypotension.

**Table 1:** Patient characteristics

**Patient number**	**Age (years)**	**Sex**	**Total IL-2 doses**	**Pre-treatment serum IL-2 levels (IU/ml)**	**Post-treatment serum IL-2 levels (IU/ml)**	**Hypotension**
**1**	49	F	11	B.D	54.4	+
**2**	63	M	10	1.1	123.2	+
**3**	24	F	8	B.D.	264.3	+
**4**	49	F	8	B.D.	130.8	+
**5**	52	M	10	B.D.	878.4	+
**6**	51	M	7	B.D.	94.6	+
**7**	70	M	7	B.D.	348.4	-
**8**	50	M	9	B.D.	226.4	+

B.D: below the limit of detection by ELISA.

### Quantitation of IL-2 in patient serum using ELISA

IL-2 concentration was measured using an ELISA kit according to the manufacturers’ instructions (eBioscience, San Diego, CA, USA). Briefly, the serum samples were added to 96 well plates precoated with an anti-IL-2 capture antibody. After overnight incubation, anti-IL-2 detection antibody conjugated with biotin was added to the wells for one hour, followed by horse radish peroxidase (HRP)-conjugated avidin for 30 min and HRP substrate for 15 min. The reaction was stopped with 1 N H_2_SO_4_, and absorbance at 450 nm was measured using an ELISA plate reader. The concentration of the samples was calculated after constructing a standard curve, and all samples were analyzed in duplicates.

### Endothelial cell cultures

Primary human pulmonary microvascular ECs (Cambrex, East Rutherford, NJ, USA) were grown on dishes pre-coated with 4 μg/ml fibronectin in DMEM containing 5% FBS. In some experiments IL-2 (100 IU/ml), TNF-alpha (20 ng/ml), or anti-IL-2 antibody (10 μg/ml; clone 5334) from R&D Systems (Minneapolis, MN, USA) were added to the culture, as described for specific experiments.

### Detection of IL-2 receptor subunits using RT-PCR and flow cytometry

The expression of IL-2Rα, Rβ, and Rγ by ECs was examined using RT-PCR. Total RNA was extracted from ECs using the RNeasy Mini Kit (Qiagen, Valencia, CA, USA). The first strand cDNA was synthesized using a High Capacity cDNA Reverse Transcription Kit (Applied Biosystem, Grand Island, NY, USA). IL-2Rα was detected using a commercially available human IL-2Rα primer pair according to the manufacturer’s instructions (R&D Systems). For IL-2Rβ and Rγ, the cDNA was denatured at 95°C for 10 min before Taq Polymerase was added. This was followed by 40 cycles of denaturation at 95°C for 60 sec, annealing at 55°C (IL-2 Rβ) or 59°C (IL-2 Rγ), extension at 72°C for 60 sec, and final extension for 7 min at 72°C. The sequences of the primers were as follows: IL-2 Rβ (F:GGCTTTTGGCTTCATCATCT; R:CTTGTCCCTCTCCAGCACTT); IL-2Rγ (F:ACGGGAACCCAGGAGACAGG; R:AGCGGCTCCGAACACGAAAC). The products of amplification were separated using a 1.5% agarose gel in 1xTBE containing 2 μg/ml ethidium bromide and were visualized using UV light. For each PCR reaction, cDNA from peripheral blood mononuclear cells and cDNA from Reference RNA (BD Biosciences, San Jose, CA, USA) were used as a positive control. The quality of the cDNA was confirmed using β-actin.

### Expression of IL-2 receptor subunits

IL-2 protein expression was examined using flow cytometry. Single cell suspensions of ECs were obtained by mechanically scraping the cells off the dishes without trypsinization. Cells were blocked with 2% FBS for 30 min, followed by incubation with anti-IL-2Rα (clone A251, BD Biosciences), anti-IL-2Rβ (clone Mikβ2, BD Biosciences), or anti-IL-2Rγ (clone TUGh4, BD Biosciences) for 30 min and washed. Cells incubated with a control isotype IgG were used to set up gating for flow cytometry analysis (BD Biosciences). Only viable cells were gated for expression of IL-2R.

### Measurement of dextran fluxes and distribution of CD144 (VE-cadherin)

Dextran fluxes across ECs were evaluated using transwell chambers in the absence of hydrostatic or oncotic pressure gradients. ECs were seeded onto transwell inserts (6.5 mm diameter and 0.4 μm pore size) coated with 4 μg/ml fibronectin. ECs were treated with paired patient serum (from patient pre- and post-IL-2 treatment samples) at the indicated times. At the same time, 250 μg FITC-conjugated dextran (10 kD) was added to the top well, and 100 μl aliquots of samples were taken from the bottom chamber at 4 and 24 hours. Each time after taking a sample, 100 μl of media was added back to the bottom chamber. For each time point, the amount of dextran in all the samples removed was measured using a fluorescence plate reader and calculated after constructing a standard curve. In all the experiments, a linear line was obtained for up to 24 hr of dextran measurement, and the slope of this linear line was calculated and referred to as permeability index (with increased permeability resulting in a greater slope, and thus greater permeability index).

### Distribution and internalization of CD144 (VE-cadherin)

To determine the distribution of CD144 (VE-cadherin) and F-actin, cells were fixed with 1% paraformaldehyde and washed with PBS. VE-cadherin was detected using a mouse anti-human VE-cadherin antibody (Clone 55-7H1, BD Biosciences) and a fluorescein-conjugated goat anti-mouse IgG. F-actin was detected using rhodamine-phalloidin. The images were acquired using a scanning confocal microscope (BioRad, Hercules, CA, USA).

To evaluate VE-cadherin endocytosis, cell surface VE-cadherin was labeled by incubating ECs at 4°C for 1 hr with an anti-human VE-cadherin antibody (10 μg/ml, clone: BV-6). Unbound antibody was removed by rinsing cells in ice-cold cell culture medium. The cells were then transferred to 37°C for 1 hr to initiate internalization of molecules from the cell surface. Cells were then fixed with 1% paraformaldehyde and incubated with a fluorescein-conjugated goat anti-mouse IgG. Images were acquired using a BioRad scanning confocal microscope. Quantification of images was performed using imagej64 software (NIH, imagej.nih.gov/ij/download/) as previously described [9].

### Statistical analysis

Data were analyzed using the Student t-test or one-way ANOVA followed by post-hoc comparisons (LSD or Bonferroni) using Prism v4.0 software (GraphPad Software, Inc., La Jolla, CA, USA). A p value less than 0.05 was considered significant. Data are expressed as the mean value + SEM.

## Results and discussion

**Figure 1:**
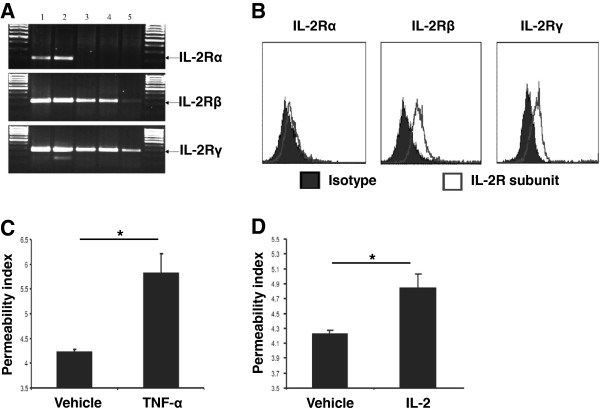
**IL-2 induces an increase in EC permeability. A)** IL-2 receptor α, β, and γ subunit levels as determined using RT-PCR. Lane 1: reference cDNA, lane 2: cDNA isolated from peripheral blood mononuclear cells, lanes 3–5: cDNA isolated from ECs from three different donors. Data are representative of five experiments with similar results. **B)** Expression of IL-2 receptor α, β, and γ subunits on the surface of ECs as examined using flow cytometry. Specific staining was compared with background staining using an isotype control antibody. Data represent three experiments with similar results. **C)** As a positive control, the effect of TNF-α (20 ng/ml) or vehicle [PBS] on EC permeability was measured by determining the flux of dextran across ECs 24 hours after treatment. **D)** ECs were treated with 100 IU/ml IL-2 or without [Vehicle: PBS], and the flux of dextran across ECs was measured 24 hours after treatment. Data in **(C-D)** are presented as mean + S.E.M. (n = 4 in each group), are representative of three experiments with similar results, and *p < 0.05 examined using ANOVA.

The mechanism by which IL-2 therapy leads to EC injury and subsequent VLS is currently unknown. We established an ex vivo pulmonary microvascular endothelial transwell flux system to explore specific changes in such cells following exposure to IL-2. This model allowed us to evaluate the flux of a small molecular weight tracer (FITC-dextran, 10 kD) across human primary ECs in the absence of a hydrostatic or oncotic pressure gradient. By setting the hydrostatic and oncotic pressure gradient constant, this system allows evaluation of EC barrier ability to restrict diffusion of dextran and to represent this value as a permeability index. To validate the model, we first confirmed expression of the trimeric IL-2 receptor on microvascular endothelial cells. We found that ECs expressed high levels of the IL-2Rβ and γ chains but only low levels of the IL-2Rα chain by both mRNA (Figure 1A) and protein (Figure 1B) analysis. This suggests that the pulmonary microvascular ECs express an intermediate affinity IL-2 receptor, and verifies previous reports [10].

Next, we determined a clinically relevant dose of IL-2 for in vitro studies by evaluating serum IL-2 concentrations in a cohort of eight patients being treated with HDIL2 (Table 1). Prior to IL-2 therapy serum IL-2 levels were undetectable (in seven patients) or low (in one patient), whereas 48 hours into HDIL2 treatment, the median serum IL-2 concentration was 178.9 IU/ml (Table 1). Based on these measurements we utilized a 100 IU/ml dose for further in vitro assays. We determined the permeability index using dextran flux across a transwell coated with the microvacular ECs, as described in the Methods. As a positive control, we utilized TNF-α, which induces EC injury via increased permeability. TNF-α significantly increased the permeability index compared to vehicle alone (5.8 ± 0.5 with TNF-α versus 4.3 ± 0.1 without TNF-α, p < 0.05) (Figure 1C). IL-2 at 100 IU/ml increased the flux of dextran across ECs (mean permeability index [mpi]: 4.8 ± 0.2 with IL-2 versus 4.2 ± 0.1 without IL-2 [vehicle], p < 0.05) (Figure 1D). Dextran flux was similar at 100, 1000, and 10000 IU/ml of IL-2 (Additional file 1: Figure S1); therefore, remaining experiments were conducted at 100 IU/ml. These data demonstrate that IL-2 alone is sufficient to increase the permeability of ECs and that changes in permeability can be detected using the transwell flux system.

**Figure 2:**
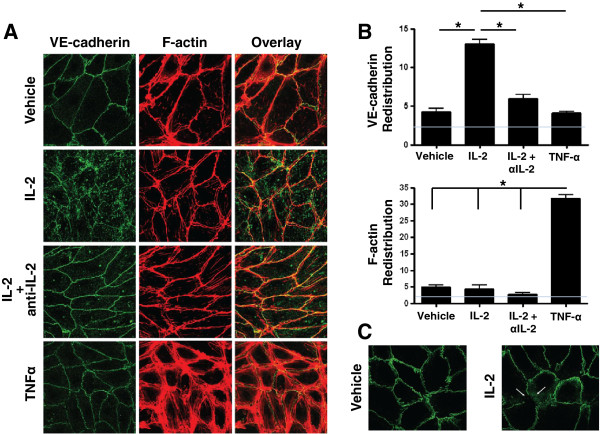
**IL-2 enhances VE-cadherin endocytosis. A)** ECs were treated with Vehicle (PBS without IL-2), 100 IU/ml IL-2, or 100 IU/ml IL-2 in the presence of a functional blocking anti-IL-2 antibody for 24 hr. VE-cadherin and F-actin were examined using confocal microscopy. The effect of TNF-α was examined as a control. **B)** Data from **(A)** were quantified using imagej64 software (NIH). VE-cadherin and F-actin intracellular redistribution (i.e., intracellular intensity in arbitrary units) is shown. Gray line indicates image background intensity. **C)** ECs were treated with Vehicle (PBS without IL-2) or 100 IU/ml IL-2 for 24 hrs. Cell surface VE-cadherin was labeled at 4°C to inhibit endocytosis. Cells were then incubated at 37°C for 1 hr to initiate endocytosis. VE-cadherin was examined using confocal microscopy. Data shown represent two independent experiments.

Changes in the EC cytoskeleton and disruption of junctional adhesion molecules have been attributed to redistribution of F-actin and VE-cadherin [11]. To determine whether such changes are involved in IL-2-mediated increases in EC permeability, we stained the ECs for F-actin and CD144 (VE-cadherin) after treatment with 100 IU/ml of IL-2 (Figure 2A). These studies demonstrated redistribution of VE-cadherin but not F-actin in the intracellular compartment (Figure 2A,B). IL-2-induced VE-cadherin intracellular redistribution was abrogated in the presence of an anti-IL-2 antibody (Figure 2A,B). Interestingly, TNF-α treatment (20 ng/ml) resulted in intracellular redistribution of F-actin but not VE-cadherin (Figure 2A,B), thus demonstrating the specificity of IL-2 action on VE-cadherin. This suggests that the action of HDIL2 on VE-cadherin may occur by a unique mechanism for vascular leak different from that which occurs in septic shock and supports blockade of VE-cadherin redistribution as a strategy for preventing IL-2-induced VLS.

The intracellular accumulation of VE-cadherin may be due to enhanced VE-cadherin endocytosis, as has been shown in the context of the vascular endothelial growth factor-induced increases in EC permeability [12]. Therefore, to determine whether IL-2-mediated redistribution was a result of VE-cadherin endocytosis, we labeled cell surface VE-cadherin (using an antibody that recognizes an extracellular VE-cadherin epitope) and transferred labeled cells to 37°C to initiate endocytosis. Increased VE-cadherin endocytosis was detected in cells treated with IL-2 compared to vehicle-treated cells (Figure 2C). These data suggest that IL-2 results in increased vascular permeability due to VE-cadherin intracellular redistribution mediated by VE-cadherin endocytosis.

**Figure 3:**
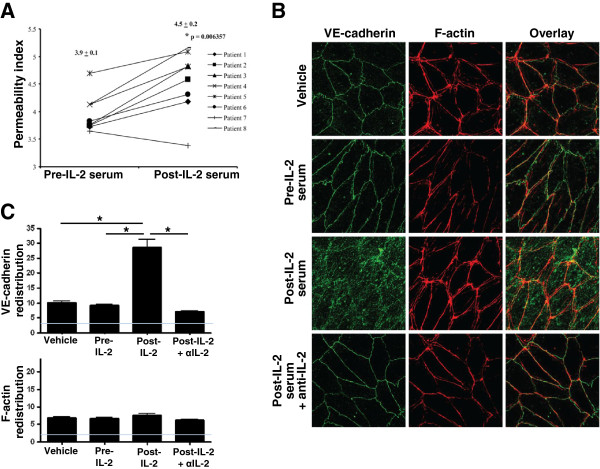
**Serum IL-2 results in altered CD144 (VE-cadherin) distribution in the EC cytoskeleton. A)** Primary human pulmonary microvascular ECs were treated with paired pre- or post-IL-2 serum, and the rate of dextran flux across an EC monolayer was measured after 24 hr of incubation. Individual data points and mean values ± S.E.M. are shown in the graph. * p was determined using paired t-test. **B)** Pre- and post-IL-2 serum was cultured with a functional blocking anti-IL-2 antibody or an isotype control IgG for 30 minutes and then used to treat ECs for 24 hr. The effect on the distribution of VE-cadherin and F-actin was examined using confocal microscopy. An image in which Patient 5 serum was used is shown and is representative of 4 patients tested. **C)** Data from **(B)** were quantified using imagej64 software (NIH). VE-cadherin and F-actin intracellular redistribution (i.e., intracellular intensity in arbitrary units) is shown. Gray line indicates image background intensity. Data represent two independent experiments.

To determine whether the observed in vitro IL-2-induced changes in VE-cadherin distribution likewise may occur in vivo, we evaluated VE-cadherin accumulation after culture of ECs with patient serum. First, we confirmed that serum from patients treated with HDIL2 significantly increased EC permeability compared with paired pre-IL-2 therapy serum in seven of eight patients (mpi: 3.9 ± 0.1 versus 4.5 ± 0.2, respectively, p = 0.006) representing an approximately 15% overall increase (Figure 3A). Interestingly, all patients in the trial developed hypotension except patient 7 (Table 1) whose EC permeability here did not increase with post-IL-2 therapy serum. Post-IL-2 serum, but not pre-IL-2 serum, induced an increase in intracellular VE-cadherin but not F-actin (Figure 3B,C). This effect was seen in post-IL-2 serum from several patients across of range of serum IL-2 concentrations (range, 130.8 - 878.4 IU/ml; see Additional file 2: Figure S2). Further, this intracellular VE-cadherin increase was abrogated by an anti-IL-2 antibody (Figure 3B,C). These data suggest that, similar to the effect of exogenous IL-2 on EC barrier function in vitro, patient serum high-dose IL-2 also has the capability to increase EC permeability through VE-cadherin redistribution.

This study provides evidence that IL-2-induced vascular leak occurs, at least in part, due to disruption of the EC barrier upon engagement of an intermediate affinity EC IL-2R and is associated with increased VE-cadherin, but not F-actin, redistribution via endocytosis. Further, this study demonstrates that IL-2 therapy-induced EC barrier dysfunction can be detected by using a novel ex vivo transwell model that measures increases in dextran fluxes across primary human pulmonary microvascular ECs. We believe that such a primary human pulmonary microvascular EC model can be used as an ex vivo methodology for future research in VLS. In our study, similar findings were made using both in vitro assays utilizing defined doses of recombinant IL-2 and ex vivo assays where ECs were exposed to sera from patients treated with HDIL2, suggesting that a similar mechanism may be operative in vivo.

## Conclusions

VLS is a major challenge to the clinical management of patients treated with HDIL2 and other related cytokines. A better understanding of the mechanisms of VLS may provide new therapeutic targets for ameliorating VLS while not interfering in the immune-mediated activity of IL-2, which is important for the therapeutic effectiveness of the cytokine treatment. In this report we identified CD144 (VE-cadherin) redistribution as a mechanism of IL-2-associated VLS, which differs from TNF-α-associated changes in vascular permeability. Other soluble and cellular mediators may also contribute to changes in vascular permeability and the transwell model may be ideal for further studies aimed at defining the mechanism of IL-2-related vascular leak syndrome.

## Abbreviations

HDIL2: High-dose IL-2; IL-2: Interleukin-2; VLS: Vascular leak syndrome; EC: Endothelial cell; VE-cadherin: Vascular endothelial cadherin.

## Competing interests

The authors declare that they have no competing interests.

## Authors’ contributions

DWK carried out the microvascular endothelial system experiments. AZ and JS carried out the VE-cadherin and F-actin distribution analyses. DWK, CR and GS contributed to the design of the experiments and sample processing. DWK, AZ and JS performed the statistical analysis. HLK conceived and designed the study. DWK, AZ, JB and HLK wrote the manuscript. All authors read and approved the final manuscript.

## Supplementary Material

Additional file 1: Figure S1 Change in dextran flux under varying IL-2 doses. ECs were treated (as described for Figure 1D) with 100. 1000, or 10000 IU/ml IL-2 or without [Vehicle: PBS], and the flux of dextran across ECs was measured 24 hours after treatment. Data are presented as mean fold change in dextran flux (IL-2-treated/Vehicle) + S.E.M. and representative of three experiments with similar results. *p < 0.05 examined using ANOVA. Click here for file

Additional file 2: Figure S2 Serum IL-2 induces CD144 (VE-cadherin) distribution. A-D) Primary human pulmonary microvascular ECs were treated (as described for Figure 3) with paired pre- or post-IL-2 serum from patient 3 (A) and patient 4 (C) and the effect on the distribution of CD144 (VE-cadherin) and F-actin was examined using confocal microscopy. (B) and (D) show quantification of data from (A) and (C), respectively, using imagej64 software (NIH). VE-cadherin and F-actin intracellular redistribution (i.e., intracellular intensity in arbitrary units) is shown. Data represent two independent experiments. Click here for file
