# Supplementary figures and images for: Interleukin-2 alters distribution of CD144 (VE-cadherin) in endothelial cells
Source: J Transl Med. 2014 May 6;12:113. doi: 10.1186/1479-5876-12-113 (PMC4062649; doi:10.1186/1479-5876-12-113)

## Additional file 1: Figure S1

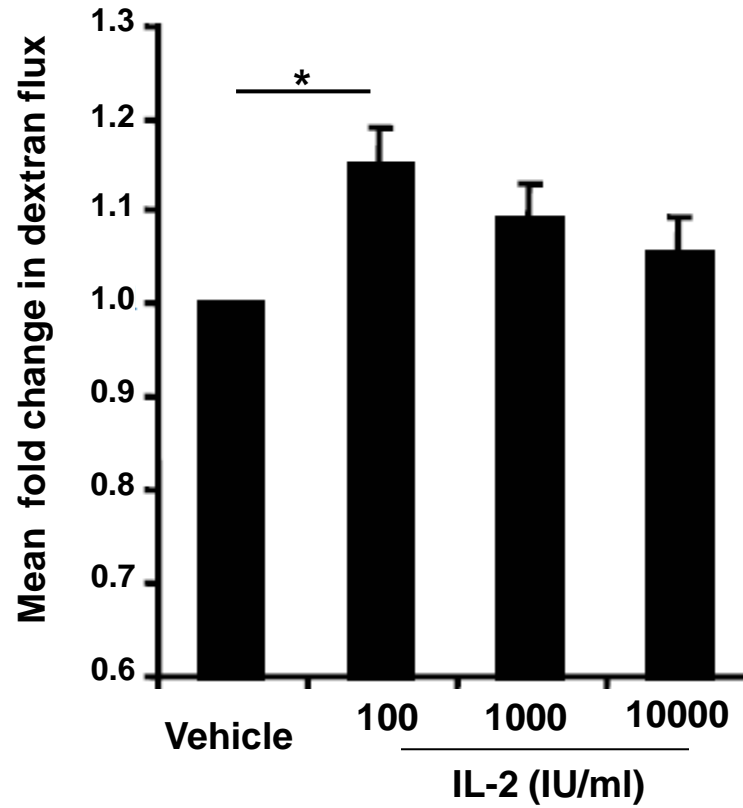

Supplement: Additional file 1: Figure S1 — Change in dextran flux under varying IL-2 doses. ECs were treated (as described for Figure 1D) with 100. 1000, or 10000 IU/ml IL-2 or without [Vehicle: PBS], and the flux of dextran across ECs was measured 24 hours after treatment. Data are presented as mean fold change in dextran flux (IL-2-treated/Vehicle) + S.E.M. and representative of three experiments with similar results. *p < 0.05 examined using ANOVA. [file 1479-5876-12-113-S1.pdf]

## Additional file 2: Figure S2

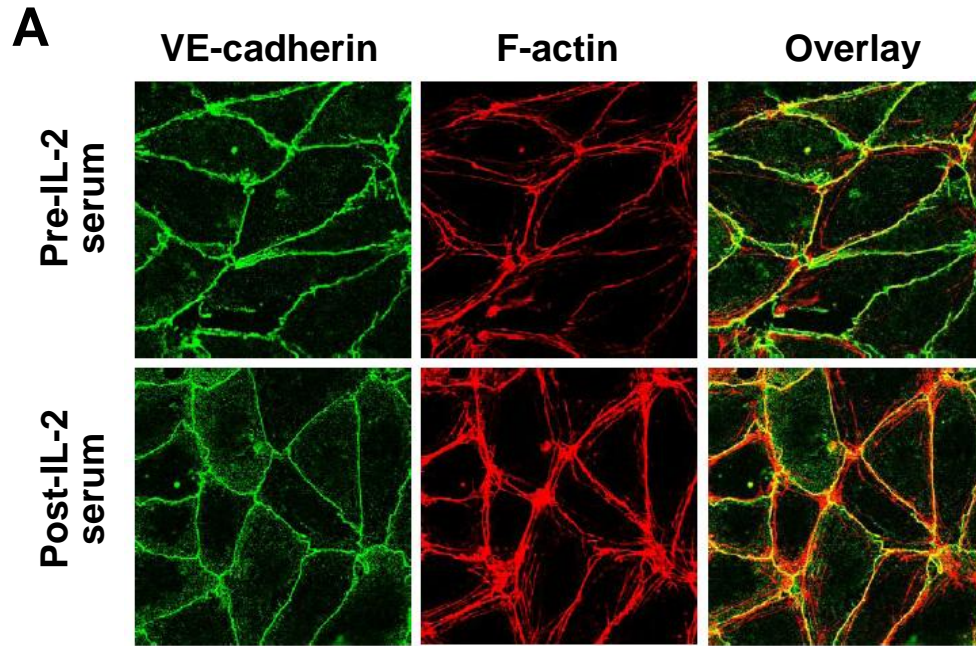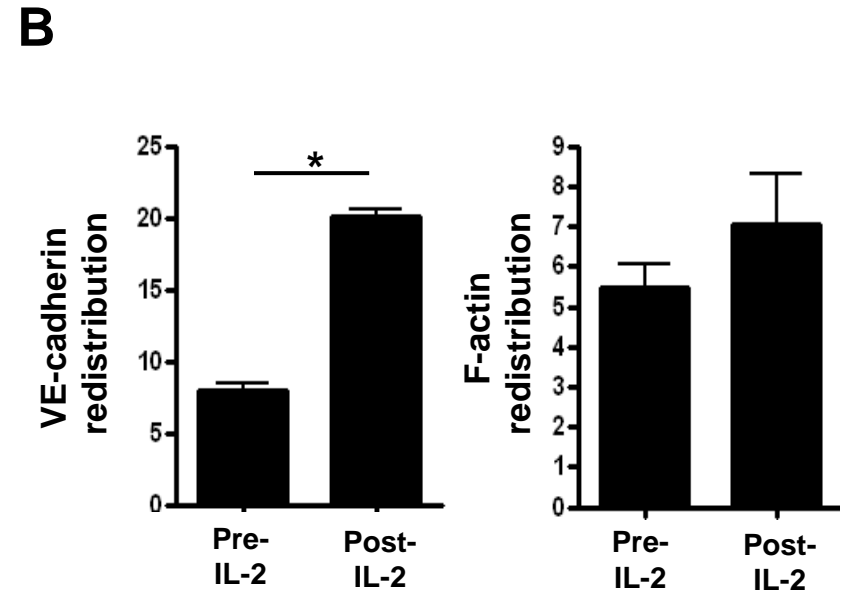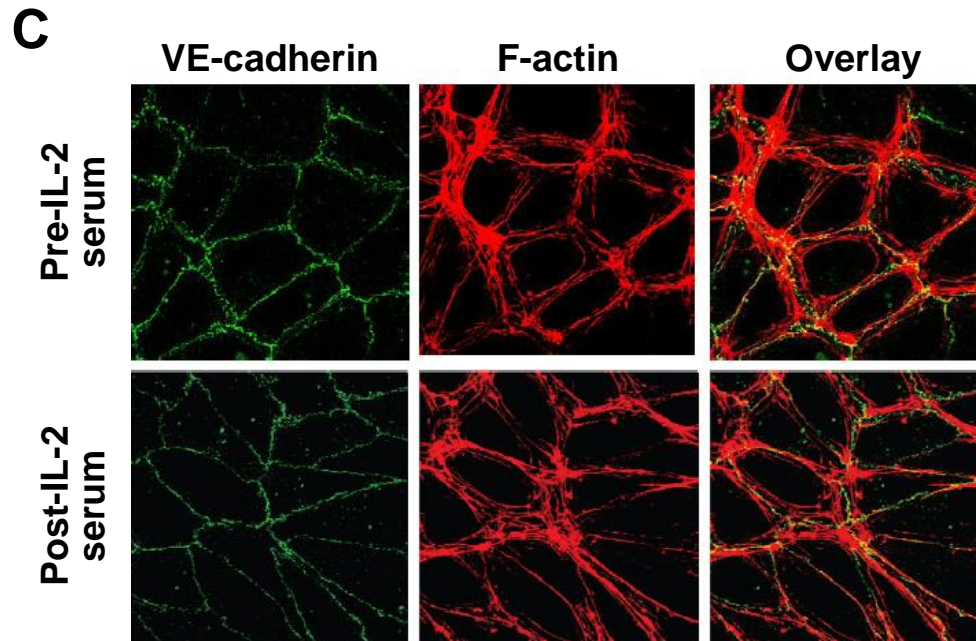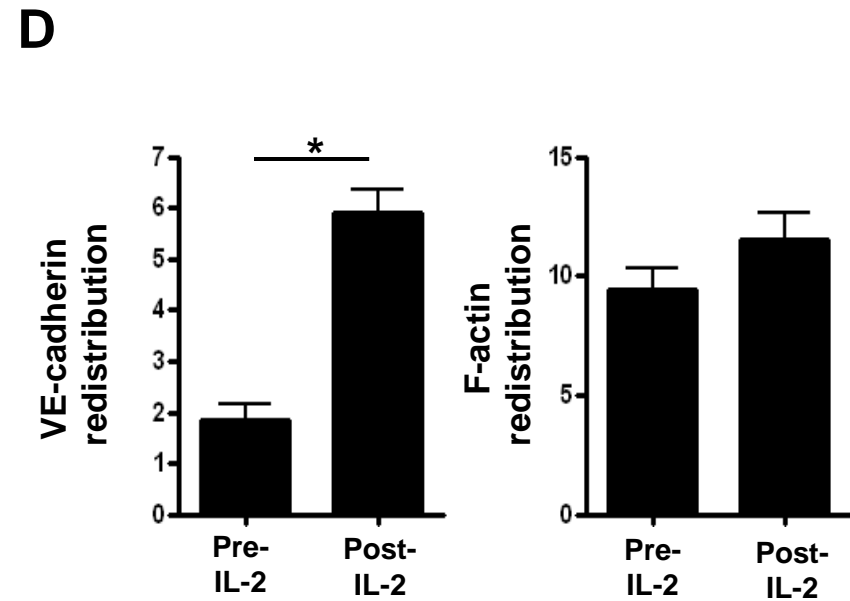

Supplement: Additional file 2: Figure S2 — Serum IL-2 induces CD144 (VE-cadherin) distribution. A-D) Primary human pulmonary microvascular ECs were treated (as described for Figure 3) with paired pre- or post-IL-2 serum from patient 3 (A) and patient 4 (C) and the effect on the distribution of CD144 (VE-cadherin) and F-actin was examined using confocal microscopy. (B) and (D) show quantification of data from (A) and (C), respectively, using imagej64 software (NIH). VE-cadherin and F-actin intracellular redistribution (i.e., intracellular intensity in arbitrary units) is shown. Data represent two independent experiments. [file 1479-5876-12-113-S2.pdf]
